# Saponins from *Quillaja saponaria* and *Quillaja brasiliensis*: Particular Chemical Characteristics and Biological Activities

**DOI:** 10.3390/molecules24010171

**Published:** 2019-01-04

**Authors:** Juliane Deise Fleck, Andresa Heemann Betti, Francini Pereira da Silva, Eduardo Artur Troian, Cristina Olivaro, Fernando Ferreira, Simone Gasparin Verza

**Affiliations:** 1 Molecular Microbiology Laboratory, Institute of Health Sciences, Feevale University, Novo Hamburgo 93525-075, RS, Brazil; julianefleck@feevale.br (J.D.F.); pereiradasilvafrancini@gmail.com (F.P.d.S.); eatroia@gmail.com (E.A.T.); 2 Bioanalysis Laboratory, Institute of Health Sciences, Feevale University, Novo Hamburgo 93525-075, RS, Brazil; andresa@feevale.br; 3 Science and Chemical Technology Department, University Center of Tacuarembó, Udelar, Tacuarembó 45000, Uruguay; cristina.olivaro@cut.edu.uy; 4 Organic Chemistry Department, Carbohydrates and Glycoconjugates Laboratory, Udelar, Mondevideo 11600, Uruguay; ff@fq.edu.uy

**Keywords:** triterpenoids, *Quillaja saponaria*, *Quillaja brasiliensis*, vaccine, immunoadjuvant, antimicrobial, antiviral

## Abstract

*Quillaja saponaria* Molina represents the main source of saponins for industrial applications. *Q. saponaria* triterpenoids have been studied for more than four decades and their relevance is due to their biological activities, especially as a vaccine adjuvant and immunostimulant, which have led to important research in the field of vaccine development. These saponins, alone or incorporated into immunostimulating complexes (ISCOMs), are able to modulate immunity by increasing antigen uptake, stimulating cytotoxic T lymphocyte production (Th1) and cytokines (Th2) in response to different antigens. Furthermore, antiviral, antifungal, antibacterial, antiparasitic, and antitumor activities are also reported as important biological properties of *Quillaja* triterpenoids. Recently, other saponins from *Q. brasiliensis* (A. St.-Hill. & Tul.) Mart. were successfully tested and showed similar chemical and biological properties to those of *Q. saponaria* barks. The aim of this manuscript is to summarize the current advances in phytochemical and pharmacological knowledge of saponins from *Quillaja* plants, including the particular chemical characteristics of these triterpenoids. The potential applications of *Quillaja* saponins to stimulate further drug discovery research will be provided.

## 1. Introduction

*Quillaja saponaria* Molina is commonly found in Peru, Chile, and Bolivia. It was first described in 1782 due to the saponin content in the bark. The expression ‘saponin’ is Latin in origin: the word sapo that means ‘soap’, since saponins form foams when mixed with water [1,2]. The amphiphilic structure of saponins, due to their lipophilic aglycones and hydrophilic saccharide side chains, is responsible for the foam formation and chemical properties [1,2]. The most commonly reported aglycone from *Q. saponaria*, i.e., quilaic acid [3,4], is characterized by an aldehyde group at the C-23 position. The acyl side chain, another characteristic of *Quillaja* saponins, seems to also play an important role in some biological activities [5].

Saponins from *Q. saponaria* have a wide range of applications. Historically, *Quillaja* bark saponins have been used as a detergent. Nowadays they are approved for use as food additives in 187 signatory countries of the Codex Alimentarius, including the European Union, the United Kingdom, the United States, China, and Japan [6], the latter of which also allows its use in cosmetics [7]. The European Commission of Cosmetic Ingredients (CosIng) database has listed some *Quillaja* products as cosmetic ingredients, such as the bark, bark extract, root extract, and wood extract. Depending on the material used, different functions are attributed, such as antidandruff, cleansing, emulsifying, foaming, masking, moisturizing, skin conditioning, and surfactant [8].

Nowadays, there is increasing interest in using saponins as natural emulsifiers, alone or blended with others. Numerous studies have reported that *Quillaja* saponins are good emulsifiers for O/W emulsions [7,9,10] and nanoemulsions [10]. Recently, a food ingredient containing *Quillaja* bark saponins (Q-Naturale^®^) was approved by the FDA as an effective emulsifier for beverages [6,10].

In addition to their detergent and emulsifying properties, other pharmacological activities, including antiviral [11,12,13], antifungal [14], antibacterial [15,16,17], and antiparasitic ones, have been reported for *Quillaja* saponins. Regarding its biological properties, *Q. saponaria* has been included in the European and Brazilian Pharmacopoeias. The raw material is recommended as a cough reliever and expectorant, anti-inflammatory, hypocholesterolemic, and hemolytic agent. Topically, it has been indicated to treat scalp diseases (dandruff and hair loss) and other dermatological disorders. In the African Pharmacopoeia, *Quillaja* bark has been indicated as an emulsifying agent only.

Meanwhile, the strong immunoadjuvant activity reported for Quil-A^®^, a crude extract of *Q. saponaria* bark, and its derivatives, makes them ideal substances for vaccine development [5,18,19,20,21]. Since Quil-A^®^ is a heterogeneous mixture of saponins, its various components may exhibit different biological properties, including toxicological effects [5]. Thus, more purified saponin fractions have been obtained, especially QS-21, which has been applied in preclinical studies and clinical trials [5,21]. 

An improvement to reduce saponin toxicity and enhance immunoadjuvant activity has been reported with the development of immunostimulating complexes (ISCOMs). ISCOMS are cage-like particles (40 nm), composed of saponins derived from *Q. saponaria*, phospholipids, cholesterol, and the targeted antigen [22,23,24]. Recently, a purified fraction of *Quillaja brasiliensis* saponins was also tested in ISCOMs, and the typical cage-like structures were viewed [25]. It is important to note that these structures are described only for *Quillaja* species. 

*Q. brasiliensis* is native to Northern Uruguay, Southern Brazil, Northeastern Argentina, and Eastern Paraguay. It has also been studied as an immunoadjuvant, with crude extracts and purified saponin fractions being evaluated and shown to stimulate a great immune response to viral antigens, as observed with saponins from *Q. saponaria* [25,26,27,28]. Saponins are also abundant in the leaves of *Q. brasiliensis*, which represents a renewable source of these substances, since in *Q. saponaria* the main source of saponins is the barks of the tree. Nowadays, the overexploitation of native forests has been replaced and other alternative sources are available such as synthetic analogues of QS-21 saponin. Meanwhile, natural alternative sources of these products are also important [29]. 

Considering the relevance and biological properties already described for *Quillaja* species, a systematic review of published trials was accomplished with respect to their chemical characteristics and biological activities. To perform the review, the PubMed database was searched using the keywords *Quillaja* saponins, chemical characteristics, biological activity, adjuvant, antiviral, antifungal, antibacterial, and toxicity, either alone or in combination.

## 2. Chemical Structure/Characterization

**Figure 1:**
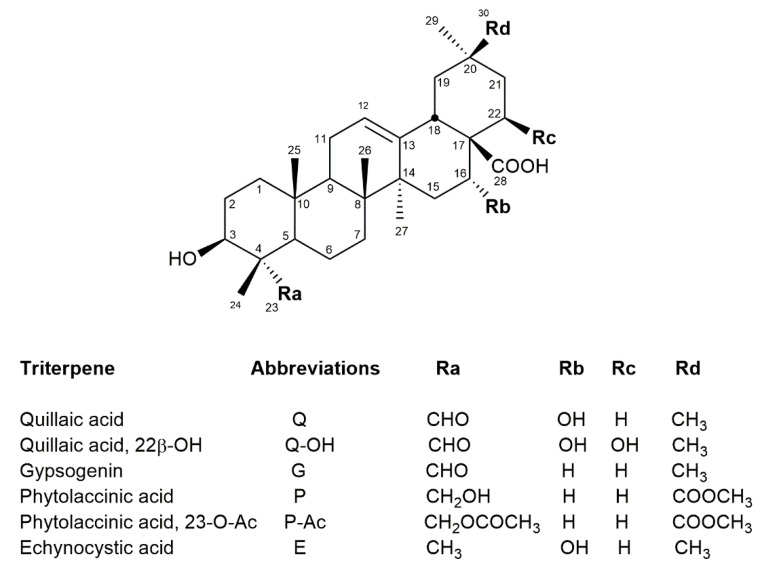
Reported triterpene aglycones in *Quillaja saponaria* and/or *Q. brasiliensis* saponins.

**Figure 2:**
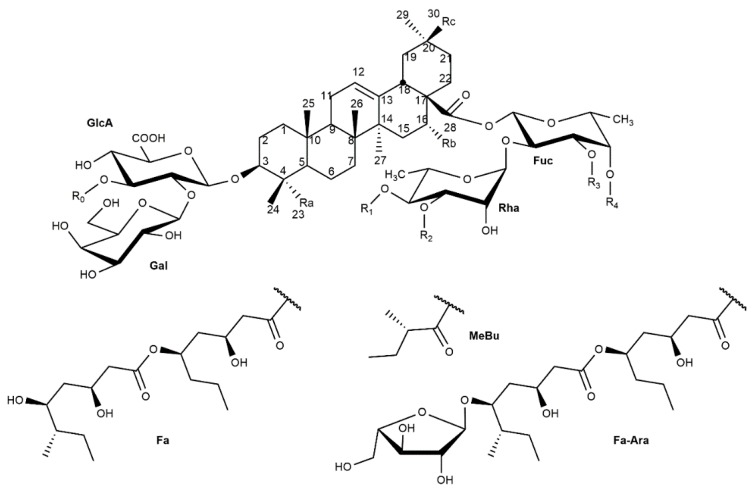
General structure of *Q. saponaria* and *Q. brasiliensis* saponins.

Despite their widespread use, the structure of *Q. saponaria* saponins was not completely elucidated until 1986, when Higuchi et al. [3] published the structure of two deacetylated saponins from *Q. saponaria* bark (Figure 1, Figure 2 and Table 1). Further isolation and structural studies allowed for the identification of nearly 60 saponins from *Q. saponaria*, sharing several structural motifs. These saponins contain a triterpenic aglycone belonging to the β-amyrin series, most frequently quillaic acid (Figure 1).

These saponins are bidesmosides, glycosylated at the C-3 and C-28 positions of the aglycone (Figure 2). The C-3 position of the aglycone is most frequently glycosylated by the disaccharide residue β-d-Gal*p*-(1→2)-β-d-GlcA*p*, most frequently branched at the O-3 position of the glucuronic acid unit with a α-l-Rha*p* or a β-d-Xyl*p* residue. In a few cases, the saponin lacks the C-28 linked oligosaccharide (saponins 3–5, Table 1), but normally this position is glycosylated through an acetal-ester linkage to a complex oligosaccharide. This oligosaccharide has a conserved moiety composed of the disaccharide α-l-Rha*p*-(1→2)-β-d-Fuc*p* with different decorations [30]. More often this disaccharide is extended by a β-d-Xyl-(1→4) residue, which can bear a second β-d-Xyl*p* (1→3) residue or a β-d-Api*f* (1→3) residue. The β-d-Fuc*p* residue of the constant disaccharide can present either a α-l-Rha*p* or a β-d-Glc*p* at O-3, and can be acetylated or not. The β-d-Fuc*p* residue can be acylated at O-4, simply with an acetate residue or a branched ester dimer composed of two units of 3,5-dihidroxy-octanoic acid, called Fa, which can be glycosylated in the terminal 5-hidroxy group by a β-d-Ara*f* residue (Figure 2) [30]. This terminal β-d-Ara*f* residue can also bear a (1→2)-β-d-Glc*p* residue.

The β-d-Fuc*p* residue linked to C-28 can be acylated at O-4 (R4 in Figure 1, see Table 1). However, the corresponding regioisomers acylated at the O-3 position can be found in solution due to a transesterification reaction between these two *cis*-vicinal hydroxyl groups, as has been observed in the adjuvant saponin QS-21. The rate of this isomerization is higher at pH > 5.5, leading to an equilibrium mixture of both isomers [31].

Phytochemical studies of the less known *Quillaja brasiliensis* tree, even though it is a well-known rich source of saponins, started relatively later. In a pioneering study, a prosapogenin composed of quillaic acid glycosylated at C-3 with a β-d-GlcA*p* residue was isolated from the products of partial hydrolysis, suggesting that these saponins might also present structures like those from *Q. saponaria* [32]. Recently, Wallace et al. [29] presented the first structural studies of complete *Q. brasiliensis* saponins using electrospray ionization ion trap-multiple stage mass spectrometry (ESI-IT-MSn). These authors identified 27 bidesmosidic triterpenic saponins, with either quillaic acid, gypsogenin, phytolaccinic acid, or 23-*O*-acetyl-phytolaccinic acid as aglycones (Figure 1). The *Q. brasiliensis* saponins described present glycosylation patterns at positions C-3 and C-28 of the aglycone analogous to those of *Q. saponaria* saponins with the same conserved motifs (Figure 2). Some saponins presented different degrees of acylation, and regioisomers with the Fa acyl substituent at the O-4 or the O-3 positions of the β-d-Fuc*p* residue were found, as well as the presence of the QS-21 Xyl and QS-21 Api saponins (S4/S6, Table 1).

## 3. Biological Activities 

Regarding the biological activities, *Quillaja* saponins have been widely studied. The main activities described are reviewed below, especially the antibacterial, antiviral, antifungal, antiparasitic, antitumor, hepatoprotective, and immunoadjuvant ones.

### 3.1. Antimicrobial Activities of Quillaja Saponins

The antimicrobial activities of saponins from *Quillaja* are described in several studies, especially their antibacterial, antifungal, and antiviral ones [11,12,14,15,38,39,40]. The main reason for these activities is the affinity of the aglycone with cell membrane cholesterol, leading to changes in the membrane and, consequently, preventing microorganism infection of the cell [41,42].

#### 3.1.1. Antibacterial Activity

Saponins present detergent properties by destroying membrane lipids and, for this reason, they can make the bacterial cell membranes permeable facilitating the influx of molecules through the bacterial wall, such as antibiotics [43,44,45]. On the other hand, a study conducted with clinical strains of multiresistant *Escherichia coli* isolated from human urine demonstrated that commercial saponin from *Q. saponaria* barks (12 μg/mL) significantly increased the growth of the six strains tested. In susceptibility tests of *E. coli* strains against ampicillin, streptomycin, and ciprofloxacin, saponins increased the colony forming units per mL (CFU/mL), even in the presence of antibiotics [46]. 

Saponin extracts from *Q. saponaria* are sold in the market and produced by different brands, and their composition can vary according to the process used to obtain them. Therefore, the antimicrobial activity may be altered since the constitution and nature of the saponins differs. Sen et al. [16] evaluated the anti *E. coli* action of *Q. saponaria* saponins from three different commercial companies (Carl Roth GmbH^®^, Karlsruhe, Germany; Sigma^®^, Saint Louis, MO, USA; Quillaja 300 Powder^®^, Nor-feed, Hvidovre, Denmark). The study showed different results between the three brands used, in which the CFU increased with the different concentrations of saponins used. The percentage of bacterial growth was 151%, 31%, and 97% for the Roth, Sigma, and Nor-feed brands, respectively. The activity of *Q. saponaria* saponins, in the induction of bacterial growth, indicates that they may act by facilitating the transport of nutrients from the bacterial growth media into the microorganism by increasing bacterial permeability though the cell membranes, thus stimulating the colonies’ growth [46,16].

In contrast, Hassan et al. [38] showed the antibacterial activity of a commercial extract rich in *Quillaja* saponins (Sigma^®^) against *Salmonella typhimurium, Staphylococcus aureus*, and *E. coli.* Similarly, Sewlikar and D’Souza [47] observed the strong antibacterial activity of the aqueous extract of *Q. saponaria* against four strains of *E. coli* producers of Shiga toxin (STEC) O157:H7 and six non-O157 STEC serotypes. At the time of treatment with the extracts, the 4 O157:H7 strains had an initial CFU of approximately 7.5 log and after 16 h of treatment, the remaining counts were reduced to 6.79 and 3.5 log CFU at room temperature. The non-O157 STECs treated with *Q. saponaria* showed reductions of 6.81 to 4.55 log CFU after 16h at room temperature. Additional tests showed that incubation at 37 °C reduced the CFU to undetectable levels (control without any significant reduction) after 1h of treatment for all O157:H7 strains and 30 min post-treatment for non-O157. Scanning electron microscopy analysis revealed the damage to bacteria cell membranes subjected to the treatment.

The commercial extract of *Q. saponaria* saponins (Sigma^®^) was shown to be a relevant agent for increasing the efficiency of the disinfection process of the bacteria genus *Asaia*. Pretreatment with 1% saponin solution resulted in increased sensitivity of the bacterial cultures tested. Cultures pre-exposed to saponin solution and submitted to *N*-ethyl-*N*,*N*-dimethylhexadecylammonium bromide (QAC) showed half of the minimum inhibitory concentration (MIC) compared to non-saponin-treated bacteria. In tests performed with peracetic acid, the difference in MICs was 4 to 8-times lower than the bacterial strains without saponin pre-treatment. With these findings, the authors suggest that treatment with saponin solution prior to disinfection may reduce the need for high concentrations of the antimicrobials tested. Concerning the inhibition of bacterial biofilms, experiments conducted in this same study revealed that the extract reduced the live cells number in biofilms by approximately 4 to 5 log [48]. Different studies on the antibacterial activity of *Q. saponaria* saponins suggest that saponins’ biological activity is related to their chemical constitution, thus different microorganisms have different susceptibilities to saponin action [49,50].

#### 3.1.2. Antiviral Activity

Two extracts of *Q. saponaria*, Ultra Dry 100 Q (a spray-dried purified aqueous extract, consisting of 65% saponins) and Vax sap (purified medical grade material, obtained by an additional purification of Ultra Dry 100 Q, with saponin content greater than 90%), showed antiviral activity in vitro and in vivo. Ultra Dry 100 Q showed strong antiviral action against herpes simplex virus type 1, vaccinia virus, human immunodeficiency viruses 1 and 2, varicella zoster virus, rhesus rotavirus (RRV), and reovirus. Very low concentrations of the extract were able to prevent the virus from infecting their host cells and were still able to maintain their blocking activity and stop the virus binding to the cells for up to 16 h after their removal from the culture medium. Both extracts studied prevented the binding of RRV and reovirus to host cells pretreated with them, also inhibiting the propagation of infectious particles to the uninfected neighboring cells. This cell protecting effect persisted for up to 16 h after the extracts were removed from the cell monolayers [11,13]. The action of saponins is possibly due to the cell membrane and the affinity of the aglycone portion with the membrane cholesterol. Consequently, without the cholesterol, the fluidity of the cell membrane increases, causing it to be beyond the control of the enzymatic activities. Thus, saponins may act in cellular proteins by preventing specific binding of viral receptors [41,51].

Confirming the evidence shown in the in vitro tests, in which the saponin-rich extracts of *Q. saponaria* Molina are able to inhibit RRV infection in host cells by some modification in the cell membrane proteins preventing binding of viral receptors, in vivo experiments with Vax sap extract presented strong anti-RRV action. Newborn mice were exposed to the aqueous extract for two days and, afterwards, RRV was administered for another five consecutive days. Doses of 0.015 mg/mouse reduced the diarrhea caused by the virus by 68% in mice exposed to 500 plaque forming units (PFU). The experiments revealed that lower concentrations of the extract do not have a strong antiviral effect when administered in higher viral titers (5000 PFU). In addition, in the case of a higher inoculant (50,000 PFU), an extract concentration of 0.03 mg/mouse blocked virus infection, also reducing diarrhea in the animals [12].

#### 3.1.3. Antifungal Activity

Membranes of regenerated cellulose nanofibers supplemented with *Q. saponaria* saponins are described as having antifungal activity. Dixit et al. [14] verified that, after 24 h, the nanofiber membranes containing dead spores of *Penicillium roguefortii* Thom caused an 80.4% grown reduction and *Aspergillus ochraceus* anamorph resulted in a 53.6% reduction. In addition, Chapagain et al. [52] described moderate activity of *Q. saponaria* bark saponins against different fungal species: *Alternaria solani* (47.5%), *Pythium ultimum* (59.12%), *Fusarium oxysporum* (56.11%), and *Verticillium dahliae* (35.92%), but not for *Colletotrichum coccodes* species.

### 3.2. Antiparasitic Activity

The saponins from *Q. saponaria* barks showed antiparasitic activity, in vitro, in a ruminal fermentation system [15,39,40]. A commercial extract of *Q. saponaria* revealed an antiparasitic effect inversely proportional to the extract concentrations tested [17].

*Quillaja* saponins were also evaluated against cloned xenic cultures of different isolates of *Blastocystis* sp., *Tetratrichomonas gallinarum,* and *Histomonas meleagridis*. The in vitro assays for antiprotozoal activity showed that the effect of this plant’s saponins are directed towards the protozoa, although more studies are necessary to elucidate the mode of action of saponins though the parasites [53].

A study performed to evaluate the effect of commercial saponins obtained from *Q. saponaria* reduced the viability of the protozoan *Trichomonas vaginalis* by 100% at a 0.025% concentration of the extract in culture medium. The aqueous extract of *Q. brasiliensis* leaves also showed anti-*T. vaginalis* activity (0.1% inhibited 98% of the microorganism’s growth). Further experiments demonstrated that saponins induced damage to the parasite membrane, suggesting that the extracts acted by inhibiting the viability of *T. vaginalis* [54]. This antiparasitic effect may be due to the detergent action of the saponins from the genus *Quillaja* in the cholesterol of the protozoa membranes, resulting in cell lysis. However, the activity may not occur in all protozoa species, since some species may not be susceptible to the toxic effects of saponins or the parasite may adapt to saponin effects [42,50,55].

### 3.3. Antitumor Activity of Quillaja Saponins

Fractions rich in *Quillaja* saponins are also described as therapeutic agents for treating and preventing cancerous diseases [56]. One of the first studies to demonstrate the saponins’ antitumor potential was conducted with Quil-A^®^, where the fraction prolonged the survival of mice with leukemia [57]. Since saponins interact with the cholesterol of cell membranes and, consequently, cause membrane damage, cell lysis, and necrosis, they present high toxicity for clinical practice. These toxic effects have been reversed by converting *Q. saponaria* fractions into stable nanoparticles, binding them to cholesterol. The QS fraction 21 (QS-21) (a more hydrophobic fraction with an acyl chain-ASAP) formulated in killing and growth inhibiting (KGI) particles selectively killed nine of the ten tumor cell lines at a concentration 30 times lower than the concentration required to kill normal monocytes. In addition, the ASAP’s lytic effect on red blood cells was inhibited by the formulation with KGI particles; cell death occurred through the apoptosis mechanism [58].

### 3.4. Hepatoprotective Activity of Quillaja Saponins

Thought-provokingly, Abdel-Reheim et al. [59] demonstrated that bark saponins from *Q. saponaria* are interesting hepatoprotective agents. In this study, the authors verified that saponin significantly decreased the elevation of ALT (achieving 57% hepatotoxicity control), AST (66%), ALP (76%), GGT (60%), NOx (77%), TC (70%), MDA (65%), LDH (54%), TG (54%), and total (54%), direct (54%), and indirect (54%) bilirubin, coupled with increased GSH (219%) and albumin (159%) levels. Furthermore, saponin from *Quillaja* bark significantly decreased liver MDA and NOx production, reinforcing that saponin has antioxidant properties, since it is able to scavenge excessive radicals and thus suppress oxidative stress [60,61]. The hepatoprotective effect of *Quillaja* bark saponin is probably due to the amelioration of oxidative stress and suppression of NOS expression, maintaining hepatocyte integrity and functioning. The decrease in TG and TC serum levels can be explained by the decrease in intestinal cholesterol absorption and by the reduction in cholesterol levels, accompanied by an increase in the HMG-CoA reductase activity and LDL receptor levels in the liver [62]. In addition, the immunohistochemical results help to explain *Quillaja* bark saponins’ protective activity, since saponins suppressed NOS expression, attenuating oxidative and nitrosative stress [59].

### 3.5. Immunoadjuvant Activity of Quillaja Saponins

One remarkable biological activity is their ability to modulate immunity towards a more cell mediated response or to enhance antibody production [18,21,63]. This property is mainly correlated with vaccinology, since saponins can act as immunoadjuvants. Despite both steroid and triterpenoid saponins from different plant species demonstrating immunogenic activity, *Quillaja* triterpenoid saponins are the most extensively studied [18]. Because of their amphiphilic structure, both aglycone and branched sugar radicals play important roles in stimulating the immune system. In contrast to other saponin structures, *Quillaja* saponins feature a fatty acid chained to β-d-Fuc*p* residue (C-28) and, at position 4, an aldehyde group, which increases their immunogenic activity leading to a more intense stimulation of the cytotoxic T lymphocytes (CTL) [20,64]. Although the immunogenic mechanism is not yet clearly understood, it is known that they can activate different pathways, including Nod-like Receptors (NLR) and Toll-like Receptors (TLR) [18,20].

Saponins can stimulate antigen-presenting cells (APC) by inducing inflammasome formation [21,65]. The inflammasome is a set of receptors and sensors that induce inflammation by activating caspase-1. Saponins can stimulate the secretion of IL-6 and IL-1β in vitro despite requiring the activation of TLR4, leading to active forms of caspase-1. Also, saponins can stimulate the expression of TLR2 interferon-α leading to active forms of IL-18 and IL-1β [66,67]. Taken together, Marty-Roix et al. (2016) found that saponins can cause an enlargement of the lymph node, indicating cell recruitment or cell proliferation through cytokine signaling [68]. The fatty acid in their structure boosts the cytotoxicity of the CD8 lymphocyte by increasing the interaction of the saponins with the cell membrane through cholesterol, inducing the formation of cell pores and, possibly, signaling to CTLs [18,19,20,69].

**Figure 3:**
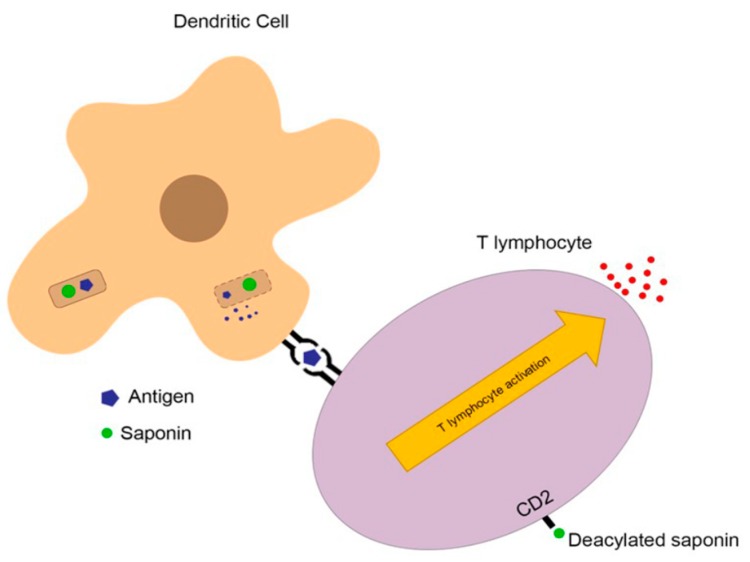
The endocytosis of the saponin with an antigen can cause disruption to the endosomal membrane of the processed antigen, facilitating the antigen presentation process by extravasation of the antigen and its transportation to the endoplasmic reticulum with posterior complexation with MHCI. Also, deacylated saponins can interact with naïve CD4 T-cells through CD2 receptors, stimulating T cell activation biased towards a Th1 response with consequent secretion of IL-2 and IFNγ.

As for the humoral response, saponins can suppress secretion of IL-4, thus lowering IgE levels. On the other hand, they can shift a Th2 response to a Th1 response, inducing the secretion of IgG2a rather than IgG1, indicating a more pronounced Th1 response than Th2 response through changes in cytokine secretion (in this context IL-2 and IFNγ) [20,70,71,72]. This mechanism may occur in two different ways: first, through the deacylated aldehyde that binds to T cell surface receptors (mostly CD2), leading the cell to Th1 immunity; and second, through endocytosis of the saponin with an antigen, disrupting the endosomal membrane of the processed antigen and facilitating the antigen presentation process (Figure 3) [20,69,73].

Despite the immunogenic activity, the considerable toxicity restricts its use in humans. An alternative for reducing saponin toxicity and increasing its immunoadjuvancy is to formulate it with cholesterol and phospholipids into cage-like structures called Immune Stimulating Complexes (ISCOMs) [74]. These structures were first described in 1971 and shown to enhance antibody production when compared to saponins, mostly because they can be easily transported to lymph nodes due to their nanoscale size (up to 80 nanometers) [75,76,77]. ISCOMs can modulate immune response in different ways according to the antigen. It is known that some antigens can increase or decrease the production of IL-10, pushing the immune response towards a Th2 or Th1 response, respectively [78,79]. ISCOMs can also stimulate CTLs through cytokine modulation [77]. The immunogenic mechanism is not clearly understood, but it is probably related to saponins, since they cause similar changes in cytokine expression, leading to different T lymphocyte activation [77,78].

In vaccine systems, saponins can be used alone or complexed with different molecules (i.e., in oil/water, in different structures (liposomes) [80], or in ISCOMs [81,82]). Also, semisynthetic saponins can be used as adjuvants in order to reduce toxicity and maintain their immunoadjuvant properties [83]. Considering their effects when used as adjuvants in vaccines, they are promising candidates and have featured in plenty of pre-clinical studies for vaccines, as mentioned in Table 2. The studies reported in Table 2 are arranged by year of publication.

According to Table 2, saponins can be formulated with a variety of antigens, either by pathogens or human proteins, and elicit a strong immune response, generally geared towards a humoral response [137]. Interestingly, over time there has been an increase in the number of studies using QS-21 rather than Quil-A^®^ or *Quillaja* extracts, probably due to its reduced toxicity [141]. Meanwhile, the chemical instability of QS-21 is reflected by acyl side chain hydrolysis, which occurs spontaneously in aqueous solutions at room temperature [142], so studies of the structure-activity relationship have been performed to enhance its adjuvant activity and reduce the undesirable effects [90,103,127]. QS-21 deacylation at Fuc-C4 was critical for Th1-type response but it was not so important for Th2-type response [90,142].

The combination of *Quillaja* saponins with other immunostimulants and formulation with carriers can take advantage of different delivery systems, such as nanoparticles [124], ISCOMs [25,86,91,96,101,113,126], ISCOM Matrix [88,113,123] and liposomes [133] for intranasal [117] or subcutaneous injections.

In Table 2 over 60 pre-clinical studies for infectious diseases, degenerative disorders, and cancer are reported. For human infectious diseases the most tested antigen was Fucose-Mannose-Ligand (FML) antigen from *Leishmania* (L.) *donovani* [90,95,97,103,106,110]. Other vaccines for infectious diseases have also been well studied, such as hepatitis B [133,137,139], malaria [113,142], and HIV-1 [21,121,126] For veterinary use, pre-clinical trials have focused on Bovine Herpes Virus-5 [27] and Bovine Viral Diarrhea Virus [25,143] Fewer studies have been reported for degenerative disorders (Alzheimer’s disease) [129,136] and oncology [116,127]. In view of the amount of studies and the promising findings, many clinical trials have been developed, as mentioned in Table 3.

Most clinical trials test the safety and efficacy of the formulations. Although Quil-A^®^ and other *Quillaja* saponins have been used in pre-clinical studies, only one study reported *Quillaja* saponins for human use [144]. In all other studies, only QS-21 has been evaluated for human use [142,144,145,146,147,148]. QS-21 has been tested in prophylactic vaccines for treatment of malaria. A phase III clinical trial was conducted to determine the efficacy, safety, and immunogenicity of the RTS/S ASO1 vaccine and received positive regulatory approval from WHO, which recommended more evaluations before high level introduction in Africa, where the clinical study was conducted. Prophylactic vaccines have been tested using gp120 protein from HIV-I [149] and hepatitis B virus surface antigen [150].

QS-21 has been used in therapeutic vaccines to treat cancer patients. In these cases, instead of being administered in healthy people, the vaccine formulation needs to induce immune responses against existing tumors [83,145,146,151,152]. A more important experiment using QS-21 in therapeutic vaccines occurred after a surgical procedure in patients in stage II melanoma. The vaccine was well tolerated, and robust antibody responses were obtained [142,145].

## 4. Toxicity of *Quillaja* Saponins

Regarding the toxicity of *Q. saponaria* and *Q. brasiliensis*, there are few studies in the literature. The studies available focus on the preclinical toxicity evaluation. One of the first studies to address the toxicity of *Q. saponaria* and its fractions was published in 1995, by Rönnberg et al. [160]. This study evaluated the toxicity of crude *Q. saponaria* extract and QH-A, QH-B, and QH-C purified triterpenoid components in WEHI cells (immature B-lymphocytes of *Mus musculus*). The evaluation of the lytic activities of *Q. saponaria*, responsible for the local reactions after injection, demonstrated that QH-A was safer than QH-B and QH-C, causing hemolysis only at a concentration ten-times higher. In addition, QH-B and QH-C did not affect RNA or protein synthesis, suggesting that they are not the primary toxicity targets. Similarly, hydrolyzed saponins of *Q. saponaria*, resulting in deacylated forms, have significantly reduced the toxicity of the substance. This supports the role of fatty acids in the toxic activity of saponins [161]. Thought-provokingly, none of the *Quillaja* saponins presented hemolytic activity after incorporation into the ISCOM matrix [160].

Although QH-B has a strong adjuvant effect, it represents the most toxic triterpenoid saponin, thus limiting its application in vitro and in vivo. Rönnberg et al. [162] demonstrated that a 10-fold higher QH-B concentration treated with sodium periodate reduced the enzymatic activities of the cells exposed to QH-B without sodium periodate by a factor of ten. The QH-B toxicity reduction is possibly the result of changes in the sugars’ chemical structure, such as xylose and galactose [160,162]. Similarly, hydrolyzed saponins of *Q. saponaria*, resulting in deacylated forms, have significantly reduced the toxicity of the substance. This supports the role of fatty acids in the toxic activity of saponins [161].

Arabski et al. [46] also studied the in vitro toxicity of *Q. saponaria*, but in the CHO-K1 cell line (cells from the ovary of *Cricetulus griseus*). They demonstrated that the saponins significantly increased death in this cell line by early apoptosis, at doses ranging from 12 to 50 μg/mL. Minor doses were not able to lyse the exposed erythrocytes, elucidating the minimal concentration required to intersperse with cell membranes in order to lyse them.

*Quillaja* saponin toxicity has also been studied in alternative mammalian models and in vivo models. Abdula et al. [163] evaluated the effects of *Q. saponaria* saponins regarding lethality and alterations in *Danio rerio* morphology during the first 3 days. This invertebrate, also known as zebrafish, is an important alternative to mammalian tests and is effective for studying toxicity mechanisms. The results showed that *Q. saponaria* presented both harmful and beneficial effects on the embryos of zebrafish, since a 5 µg/mL concentration promoted growth and a 10 µg/mL concentration was mortal to zebrafish. These findings suggest that the toxic effects of *Q. saponaria* saponins could affect the semipermeable protective chorion expressed by the embryo in development. Alternatively, it could affect cells in the embryo bodies, by permeating the noncellular chorion.

The toxicity of *Quillaja* extracts and their saponin derivatives was also studied in neonates of the aquatic crustacean *D. magna* and in *D. rerio* zebrafish embryos [164]. The authors demonstrated that *Quillaja* extract and *Q. saponaria* fractions QS-18 (35.1 ± 4.1% of the total *Quillaja* saponins) and QS-21 (3.7 ± 0.3%) have similar toxicity; nevertheless, with a toxicity potential three to eight times higher than non-saponins. Thus, *Quillaja* saponins contribute to the majority of the toxicity (85.1–93.6%), with QS-21 being slightly more toxic than QS-18. The non-saponins contribute to only 14 and 6%. The higher lipophilicity of QS-18 and QS-21 is probably responsible for their higher toxicity, since lipophilic substances can easily cross the embryo chorion, resulting in a higher uptake in zebrafish embryos [165]. 

In vivo *Quillaja* extract acute toxicity was studied by Tam and Roner [8]. They orally treated newborn mice with 50 μL of saponin extract. The concentrations ranged from 0 to 0.5 mg/mouse. The animals were treated for seven days and observed for two months. The 0.0375 mg/mouse dose presented more than 50% mortality, thus the LD_50_ of the extract was established at 0.0325 mg/mouse. The weight evaluation demonstrated that there was no short-term health impact in mice that survived treatment with saponins. On the other hand, the high concentrations of the extract killed the mice within 24–36 h, suggesting an important alteration in the membrane environment of the gastrointestinal tract, leading cells to burst.

Finally, Vinai et al. [166] studied the safe dose of vaccine adjuvants, such as *Quillaja* saponins, for intra-peritoneal vaccination. Flounder fingerlings were acutely treated and lethality and toxicity signs, such as body color, feeding, and swimming behavior, were evaluated. The group treated with saponins showed different toxicity levels, according to the concentrations tested. The toxicity signs observed were fish lethargy, body color alterations, and alterations in feeding behavior; the liver and intestine presented important hemorrhages, the fish showed pale gills, and the peritoneal cavity was filled with red ascites, suggesting high toxicity of the highest dose tested. In this study, *Quillaja* saponins presented a LD_50_ of 105 μg/fish (22.4 mg/kg), reinforcing the high toxicity. In addition, they caused severe histological damage, as well as liver damage, observed by the increase in AST level at doses above 16 μg/fish and of ALT at 160 μg/fish.

In clinical studies only QS-21 toxicity have been studied. For most patients the dose-limiting toxicity is about 50 µg/dose, except for cancer patients (100–200 µg per vaccination) [142]. Therefore, the safety of *Quillaja* saponins is still not well established and more studies are necessary.

## 5. Conclusions

*Quillaja* saponins have been employed in the food industry as emulsifiers for beverages and food additives. In the cosmetic industry, they have been used as antidandruff, cleansing, emulsifying, foaming, masking, moisturizing, skin conditioning, and surfactant agents. However, these are not the only applications of *Quillaja* saponins. The particular chemical characteristics of these metabolites, along with their biological activities, show potential for drug discovery and vaccines development. Most of these saponins are bidesmosides and contain a triterpenic aglycone belonging to the b-amyrin series, most frequently quillaic acid. Vaccine adjuvation is the main propriety studied for *Quillaja* saponins, which have shown potent immunogenic activity and the ability to modulate immunity towards a cell-mediated response or to enhance antibody production. In addition to improving immunoadjuvant activity and minimizing their toxicity, these saponins are been evaluated in different formulations, such as ISCOMs, oil-in-water formulations, and liposomes, among others.

Furthermore, studies have demonstrated antiviral (herpes simplex virus type 1, human immunodeficiency viruses 1 and 2, varicella zoster virus, vaccinia virus, rhesus rotavirus, and reovirus), antifungal (*Penicillium roguefortii*, *Aspergillus ochraceus*, *Alternaria solani*, *Fusarium oxysporum*, *Verticillium dahlia*, and *Pythium ultimum*), and antibacterial (*Staphylococcus aureus*, *Salmonella typhimurium*, and *Asaia* spp.) activities for *Quillaja* saponins. However, their activity against *E. coli* is not established, since different commercial extracts of *Quillaja* saponins have shown opposing results. Studies have also demonstrated antiparasitic (in vitro), antitumor (in vitro and in vivo), and hepatoprotective (in vivo) activities. Regarding toxicity, there are few studies in the literature, which focuses on evaluating preclinical toxicity and lacks clinical studies.

## Figures and Tables

**Table 1:** Structures of *Quillaja saponaria* saponins.

Nº	Saponin	Mi	Structure						Ref.
			Aglycone	R0	R1	R2	R3	R4	
1	DS-1	1512.0	Q	Xyl	Api-Xyl	H	H		[3]
2	DS-2	1674.0	Q	Xyl	Api-Xyl	H	Glc		[3]
3	*	824.0	Q	H	-	-	-	-	[33]
4	*	970.0	Q	Rha	-	-	-	-	[33]
5	*	956.0	Q	Xyl	-	-	-	-	[34]
6	4	1436.6	Q	H	Xyl	H	Rha	Ac	[34]
7	5	1582.7	Q	Rha	Xyl	H	Rha	Ac	[34]
8	6	1568.7	Q	Xyl	Xyl	H	Rha	Ac	[34]
9	7	1714.7	Q	Rha	Xyl-Api	H	Rha	Ac	[34]
10	8	1700.7	Q	Xyl	Xyl-Api	H	Rha	Ac	[34]
11	9	1714.7	Q	Rha	Xyl-Xyl	H	Rha	Ac	[34]
12	10	1700.7	Q	Xyl	Xyl-Xyl	H	Rha	Ac	[34]
13	11a	1598.7	Q	Rha	Xyl	H	Glc	Ac	[35]
14	11b	1584.7	Q	Xyl	Xyl	H	Glc	Ac	[35]
15	12a	1628.7	Q	Rha	H	Glc	Glc	Ac	[35]
16	12b	1614.7	Q	Xyl	H	Glc	Glc	Ac	[35]
17	13a	1730.7	Q	Rha	Xyl-Api	Glc	H	Ac	[35]
18	13b	1716.7	Q	Xyl	Xyl-Api	Glc	H	Ac	[35]
19	14a	1760.8	Q	Rha	Xyl	Glc	Glc	Ac	[35]
20	14b	1746.7	Q	Xyl	Xyl	Glc	Glc	Ac	[35]
21	15a	1640.7	Q	Rha	Xyl	H	Glc-Ac	Ac	[35]
22	15b	1626.7	Q	Xyl	Xyl	H	Glc-Ac	Ac	[35]
23	16a	1802.8	Q	Rha	Xyl	Glc	Glc-Ac	Ac	[35]
24	16b	1788.7	Q	Xyl	Xyl	Glc	Glc-Ac	Ac	[35]
25	17a	1744.8	Q	Rha	Xyl	Glc	Rha	Ac	[35]
26	17b	1730.7	Q	Xyl	Xyl	Glc	Rha	Ac	[35]
27	18a	1876.8	Q	Rha	Xyl-Api	Glc	Rha	Ac	[35]
28	18b	1862.8	Q	Xyl	Xyl-Api	Glc	Rha	Ac	[35]
29	S1	1870.9	Q	Rha	Xyl	H	H	Fa-Ara	[36]
30	S2	1856.9	Q	Xyl	Xyl	H	H	Fa-Ara	[36]
31	S3	2002.9	Q	Rha	Xyl-Xyl	H	H	Fa-Ara	[36]
32	S4	1988.9	Q	Xyl	Xyl-Xyl	H	H	Fa-Ara	[36]
33	S5	2002.9	Q	Rha	Xyl-Api	H	H	Fa-Ara	[36]
34	S6	1988.9	Q	Xyl	Xyl-Api	H	H	Fa-Ara	[36]
35	S7	1912.9	Q	Rha	Xyl	H	Ac	Fa-Ara	[4]
36	S8	1898.9	Q	Xyl	Xyl	H	Ac	Fa-Ara	[4]
37	S9	2045.0	Q	Rha	Xyl-Xyl	H	Ac	Fa-Ara	[4]
38	S10	2030.9	Q	Xyl	Xyl-Xyl	H	Ac	Fa-Ara	[4]
39	S11	2045.0	Q	Rha	Xyl-Api	H	Ac	Fa-Ara	[4]
40	S12	2030.9	Q	Xyl	Xyl-Api	H	Ac	Fa-Ara	[4]
41	B1	2033.0	Q	Rha	Xyl	Glc	H	Fa-Ara	[36]
42	B2	2018.9	Q	Xyl	Xyl	Glc	H	Fa-Ara	[36]
43	B3	2165.0	Q	Rha	Xyl-Api	Glc	H	Fa-Ara	[36]
44	B4	2151.0	Q	Xyl	Xyl-Api	Glc	H	Fa-Ara	[36]
45	B5	2165.0	Q	Rha	Xyl-Xyl	Glc	H	Fa-Ara	[37]
46	B6	2151.0	Q	Xyl	Xyl-Xyl	Glc	H	Fa-Ara	[37]
47	B7	1886.9	Q	H	Xyl	Glc	H	Fa-Ara	[37]
48	B8	2018.9	Q	H	Xyl-Api	Glc	H	Fa-Ara	[37]
49	QS-III	2297.0	Q	Xyl	Xyl-Api	Glc	Fa-Ara-Rha	H	[3]
50	20a	1656.7	Q-OH	Rha	Xyl	Glc	H	MeBu	[34]
51	20b	1642.7	Q-OH	Xyl	Xyl	Glc	H	MeBu	[34]
52	21a	1788.8	Q-OH	Rha	Xyl-Api	Glc	H	MeBu	[34]
53	21b	1774.8	Q-OH	Xyl	Xyl-Api	Glc	H	MeBu	[34]
54	22a	1978.9	Q-OH	Rha	Xyl-Api	Glc	Rha	OHMeHex	[34]
55	22b	1964.9	Q-OH	Xyl	Xyl-Api	Glc	Rha	OHMeHex	[34]
56	19	1392.7	P	H	H	H	Glc	MeBu	[34]
57	23	1732.8	E	Xyl	Xyl	Glc	Glc	Ac	[34]
58	S13	1560.7	P-Ac	H	H	MeBu	Glc	MeBu	[4]

*: Monodesmosidic C-3 saponins. DS: deacylateded saponins, Q: quillaic acid, Q-OH: quillaic acid, 22β-OH, P: phytolaccinic acid, P-Ac: phytolaccinic acid, 23-*O*-Ac, E: echynocystic acid. Fa-Ara: see Figure 2, QS_III: quillaja saponin with highest molecular mass, MeBu: 2-methylbutanoyl, OHMeHex: 3-hydroxy-4-methylhexanoyl. Adapted from Kite et al. [30].

**Table 2:** Pre-clinical studies using *Quillaja* saponins as an adjuvant in vaccine formulations targeting different antigens.

Antigen	Adjuvant	Model	Findings	Ref.
PorA P1.6 (membrane porin of *Neisseria meningitidis*)	Quil-A^®^	Mice	PorA supplemented with Quil-A^®^ resulted in four to seven times higher antibody titers (IgG1, IgG2a, and IgG2b) when compared with lipopolysaccharide as an adjuvant.	[84]
*S. bovis* and *Lactobacillus* spp.	Quil-A^®^	Cattle	Immunization of feedlot steers against S bovis and Lactobacillus spp with vaccines incorporating Freund adjuvant, Quil-A^®^, dextran, or alum as an adjuvant effectively induced high IgG concentrations when dextran was used as an adjuvant.	[85]
38-kDa mycobacterial protein	ISCOMs	Mice	An increase in antibody titers was observed after a booster injection, with significant levels of IgG2a. The ISCOM formulations induced this in the same way. Regarding the induction of CTL responses, the differences shown comparing various ISCOMS formulations were minimal.	[86]
Tetanus toxoid	QS-21	Mice	Administration of QS-21 p.o. as an adjuvant elicits strong serum IgM and IgG Ab responses.	[87]
Babesian equi immunogen	Quil-A^®^	Donkeys	A mixture of Quil A^®^ and Babesia equi immunogen was optimal in generating a significant immune response and reducing the lethality.	[88]
OVA	QS-21 and deacylated QS-21 (DS-1)	Mice	Despite DS-1 requiring a higher dose to induce IgG1 responses, it did not induce IgG2a or CTL responses. Lower doses of QS-21 induced higher IgG titers, including IgG2a and CTL responses.	[89]
FML *	Quil-A^®^	Dogs	The formulation protected dogs against canine kala-azar in the field with robust immunogenicity.	[90]
Measles virus	ISCOMs QA-22	Rats and Macaques	Vaccines induced high levels of antibodies, which showed no decrease during a 2-year follow up.	[91]
Herpes virus	*Quillaja* saponins	Mice	Saponins’ deacylation significantly reduced antibody production and increased mortality rates during viral challenge test.	[92]
Opa J from *Neisseria meningitidis*	Quil-A^®^	Mice	Quil-A^®^ formulated with OpaJ was highly immunogenic, inducing the production of Opa-specific antibodies.	[93]
Non-fimbrial adhesin hemagglutinin B (HagB)	Semi-synthetic saponin of *Quillaja* GPI-0100	Mice	GPI-0100 showed better immunoadjuvancy than monophosphoryl lipid A and alum, as a mucosal and systemic adjuvant, in inducing serum anti-HagB.	[94]
FML *	Quil-A^®^	Dogs	The formulation was shown be effective in immunotherapy against visceral leishmaniasis of asymptomatic infected dogs. All animals showed significantly increased CD8 lymphocyte percentages.	[95]
Human respiratory syncytial virus (HRSV)	HRSV-ISCOMs prepared with *Quillaja* saponin fractions: QH-A; QH-C; QH-A + QH-C (QH-AC)	Mice	In general, the ISCOMs tested were well tolerated. However, the combination of QH-A + QH-C ISCOMs was lethal in neonates despite the QH-A or QH-C fractions alone being well tolerated.	[96]
FML *	*Quillaja saponaria* sapogenins containing aldehyde	Mice	Vaccines elicited high levels of antibodies and cellular specific response to FML and IFNγ sera levels and protection against *L. donovani* murine infection was shown.	[97]
Ovalbumin (OVA)	Quil-A^®^	Mice	Increasing amounts of Quil-A^®^ (20% to 70%) were tested in liposomes. Higher doses of Quil A reduced the particle sizes formed, thus decreasing antigen incorporation and uptake by DC. Liposomes containing 20% Quil-A^®^ were more effective as immunostimulants, and more toxic in cell cultures too, when compared with those containing 70% Quil-A^®^.	[98]
Mtb72F (*Mycobacterium tuberculosis*)	AS02A	Mice	Increase in both Th1 and Th2 response, with Th1 response being more pronounced.	[99]
LSP *Pb*CS 242–310 **	QS-21	Mice	The use of LSP *Pb*CS 242–310 combined with QS-21 induced a satisfactory immune response similar to the one generated with the injection of radiation-attenuated sporozoites.	[100]
HRSV	ISCOMs formulated with *Quillaja* saponin fractions	Mice	All three formulations favor Th1 responses to different degrees with IFN-c being produced up to 50 times more than Il-4 and IL-5. The HRSV 703 ISCOMs induced the most pronounced innate and acquired response with the most prominent Th1 profile.	[101]
HIV-1 DNA prime/protein–Env and Gag ***	QS-21	*Rhesus* macaques and mice	Cellular and humoral responses were observed when Polyvalent DNA prime/protein boost vaccine was administered. CD8^+^ CTL, CD4^+^ T-helper cells and Th1 cytokines were involved in cellular immune response.	[102]
FML *	Fractions of the Riedel de Haen saponin mixture (QS-21 saponin fraction; two deacylsaponins mixture; a mixture of glucose, rutin, quercetin, and sucrose).	Mice	QS-21 and the deacylsaponins induced the most significant reduction of parasite burden in the liver, demonstrating a promising adjuvant potential in the Riedel de Haen saponin mixture, which contains deacylated saponins that are not toxic and induce robust immunity.	[103]
Plant-made measles virus hemagglutinin (MV-H) protein	Cholera toxin (CTB/CT); LT(R192G) ****; Saponin extracted from *Quillaja* bark (Sigma; S-4521)	Mice	Despite both LT(R192G) and the crude saponins showing strong adjuvant activity, the crude extract had superior immunostimulatory properties.	[104]
Fh15 (recombinant 15 kDa *Fasciola hepatica* protein)	ADAD System *****	Mice and sheep	Mice immunized with ADAD system with Qs had a survival rate of 50–62.5% while animals without Qs had a survival rate of 40–50%. Sheep immunized with ADAD system with Qs showed less hepatic damage compared to control group.	[105]
FML *	QS-21	Dogs	Despite nonspecific reactions being observed in the immunized animals (similar to other veterinary vaccines), a significant decrease was shown with subsequent doses.	[106]
LTB-ESAT-6 ⬪; and Bacillus Calmette-Guérin (BCG)	Quillaja extract	Mice	Mice vaccinated with a combination of plant-made LTB-ESAT-6 fusion BCG oral adjuvant had significantly more IL-10 production when compared with mice vaccinated without adjuvant. However, no protection was shown during the challenge test with M. tuberculosis, in groups treated with oral BCG (with or without saponin).	[107]
GD3-KLH	GPI-0100 ⬪⬪; sQS-21 ⬪⬪⬪	Mice	Both synthetic *Quillaja* saponin molecules produced antibodies in mice.	[108]
FALVAC-1A (different epitopes from *Plasmodium falciparum*)	AIPO4, QS-21, Montanide ISA-720, or CRL-1005	Mice	QS-21 was the second adjuvant that induced the most significant levels of antibodies, inducing predominantly IgG2c. QS-21 also induced the highest levels of IL-4 compared to the other adjuvants, indicating Th1 and Th2 responses.	[109]
FML *	QS-21	Dogs	The vaccine induced robust immunogenicity with high levels of FML-seroconversion, as demonstrated by CD8+ and CD4+ T cell populations.	[110]
AD-472 ⬪⬪⬪⬪ or HSV-2 glycoprotein D	GPI-0100 ⬪⬪	Guinea pigs	Both formulations reduced clinical disease; however, GPI-0100 improved the glycoprotein-D formulation only.	[111]
H9N2	Aluminium phosphate; aluminium hydroxide; MF59 ⬪⬪⬪⬪⬪; and MATRIX-M^#^	Mice	MATRIX-M shifted the immune response to an IgG2a response (towards a Th1 response). On the other hand, the CD8^+^ T-cell response could be improved using MATRIX-M or MF59. Among all tested molecules, MATRIX-M was the most effective in inducing the immune response, followed by MF59 and aluminium-based adjuvants.	[112]
HpaA and catalase	ISCOMATRIX^TM^; ISCOM^TM^; Cholera toxin; and aluminium hydroxide	Mice	ISCOMATRIXTM and ISCOMTM vaccines, using two different antigens and different delivery systems (intranasal or subcutaneous), have the same efficacy in reducing *H. pylori* colonization as the gold standard cholera toxin (CT) adjuvant	[113]
TA-CIN ^##^	GPI-0100 ⬪⬪	Mice and monkeys	Prophylactic vaccination with adjuvanted TA-CIN protected the mice from viral challenge, whereas vaccination without adjuvant was almost ineffective. Moreover, GPI-0100 boosted IFN-γ secreting CD8^+^ T cell response in mice compared to the formulation without adjuvant. Vaccination of macaques induced specific T cell responses.	[114]
Inactivated Chlamydophila abortus	QS-21	Mice	The formulation induced the proliferation of B cells, which is important to confer immunity to the bacteria rather than cellular response.	[115]
sLE^a^-KLH (glycolipid/glycoprotein expressed on cancer cell surface)	GP1-0100	Mice	Immunized animals produced high titers of highly reactive IgM and IgG specific antibodies.	[116]
Tetanus toxoid	Quillaja saponins or cross-linked dextran microspheres (CDM)	Rabbits	Formulations with Quillaja saponins did not increase mucosal IgA. On the other hand, the saponins induced the highest systemic IgG titers.	[117]
Ovalbumin	ISCOM matrices	Mice	The formulation with ISCOMs induced high specificity CTLs targeting different tumoral cells.	[118]
Tetanus toxoid loaded onto PLGA nanospheres	Quillaja saponins, CDM	Rabbits	The combination of Quillaja saponins with CDM in PLGA nanospheres loaded with antigens improved IgA production, suggesting better mucosal protection. The same was observed with systemic IgG.	[119]
Inactivated Bovine herpesvirus-5	Aluminum hydroxide, Quil-A^®^, or QB-90 (from Quillaja brasiliensis)	Mice	Quil-A^®^ and QB-90 induced similar immunity regarding humoral or cellular responses.	[27]
ALM (Leishmania major)	PLGA nanoparticles containing purified saponin extract	Mice	Increase in both Th1 andTh2 response.	[120]
HIV-1 gp120 with Q105N mutation	Quil-A^®^ or MPL	Mice	Despite both adjuvants producing specific antibodies, there was no significant neutralizing activity against HIV-1.	[121]
Tetanus toxoid	Quillaja saponins	Rabbits	Quillaja formulation was more efficient in conferring mucosal protection due to increased levels of IgA.	[122]
No antigen was used	Alhydrogel or MATRIX-M^TM^ formulated with fractions of Q. saponaria	Mice	Besides stimuli caused by MATRIX-M^TM^ during the immune response (increase in cell recruitment and cytokine secretion), it induced the expression of co-stimulatory molecule CD86, participating in early events of the immune response.	[123]
Rhoptry Toxoplasma gondii protein	Quil-A^®^	Swine	The formulation induced high humoral, local, and systemic immune response, partially preventing brains from forming cysts.	[124]
Tetanus toxoid loaded onto chitosan functionalized gold nanoparticles	Quillaja saponins	Mice	When administered orally, the nanoparticles can better deliver the formulation, increasing immune response up to 28-fold compared to control.	[125]
HIV-1 gp120	QS-21, aluminum hydroxide, MPLA, or ISCOMATRIX^TM^	Mice	Each adjuvant stimulated a different cytokine profile secretion, having different properties in the induction of the inflammation.	[126]
MUC1-KLH (prostate cancer marker) and/or ovalbumin	QS-21, synthetic QS-21, or other conjugated saponins	Mice	Despite QS-21 inducing robust Th1 and Th2 response, conjugated saponins had improved activity and toxicity profiles relative to QS-21.	[127]
*Varicella zoster* glycoprotein E and ovalbumin	AS01	Mice	AS01 can improve adaptive humoral response through the generation of a great number of antigen-presenting cells.	[128]
Inactivated *Poliovirus*	Quil-A^®^, QB-90, and Aqueous Extract from *Quillaja brasiliensis*	Mice	The humoral enhancements caused by Quil-A^®^ and QB-90 were statistically similar. However, the mucosal immune response was increased with QB-90 by increases in IgA.	[26]
Tau antigen (associated with Alzheimer’s disease)	Quil-A^®^	Mice	Animals that received the formulations with Quil-A^®^ had reduced neuroinflammation and tau pathogenesis.	[129]
MARV-VLP (*Marburg virus* virus-like particle containing glycoprotein, matrix vp40, and nucleoprotein)	QS-21 or polyI:C	Cynomolgus macaques	Formulations prepared with either of the adjuvants provided full protection in the challenge test. QS-21 produced a lower response to antigens compared to polyI:C.	[130]
EBOVgp-Fc (*Ebola virus* glycoprotein fused to Fc fragment of human IgG1)	QS-21, aluminum hydroxide, or poly-ICLC	Guinea pigs	All formulations induced antibody formation, however, despite QS-21 inducing a strong humoral response, in the challenge test only poly-ICLC induced robust protection.	[131]
Intanza 2013 (Trivalent Influenza Vaccine, Sanofi Pasteur)	QS-21	Mice	When administered in the skin as a Nanopatch vaccine, a lower dose of QS-21 and adjuvant was required to enhance humoral response compared to intramuscular administration.	[132]
Ovalbumin or HBsAg (*Hepatitis B virus* surface antigen)	QS-21 liposome	Mice	The immunoadjuvancy of QS-21 relies on macrophages to induce activation of dendritic cells and stimulate the immune system.	[133]
FhSAP2-IBs (*Fasciola hepatica* protein)	QS-21 or Montanide ISA720	Mice	Increase in both Th1 and Th2 responses with significant increase in Th1 response.	[134]
QS-21 or QS-21 formulated with HIV-1 gp120	QS-21	Mice	QS-21 activates NLRP3 inflammasome.	[21]
Inactivated bovine viral diarrhea virus	Saponins of *Q. brasiliensis* (QB-90) and *Q. brasiliensis* aqueous extract (AE)	Mice	In vaccines adjuvanted with QB-90 and AE higher levels of antibody were detected. Animals that received QB-90 adjuvanted vaccine had enhanced cytokines and IFN-γ production by CD4+andCD8+T lymphocytes whereas AE-adjuvanted preparation stimulated humoral response only.	[135]
Ovalbumin	ISCOMs of *Q. brasiliensis* saponin fraction (IQB-90) and Quil-A^®^ ISCOMs	Mice	IQB-90 inoculated subcutaneously induced strong antibody response (IgG1and IgG2a) and increased Th1 response. IQ-90 delivered intranasally induced secretion of serum IgG and IgG1 and mucosal IgA.	[25]
No antigen was used	MATRIX-M^TM^	Swine	MATRIX-M^TM^ formulation induced pro-inflammatory cytokines, suggesting enhancement of innate immune response of specific pathogen free pigs exposed to reared pigs.	[68]
Aβ42 (Alzheimer’s disease pathological hallmark)	QS-21	Rhesus monkeys	The formulation induced a good humoral response with high titers of IgG and IgA. No inflammatory cellular immune response was observed.	[136]
Ovalbumin and HBsAg (*Hepatitis B virus* surface antigen)	QS-21	In vitro (THP-1 cell) and mice	Human monocyte-derived dendritic cells were directly activated by QS-21, requiring cathepsin B to induce high CD4 and CD8 T cell response.	[137]
FMP013 (Falsiparum Malaria Protein-013 from *Plasmodium falciparum*)	ALF, ALF + aluminum hydroxide, ALFQ + QS-21, or Montanide ISA720	Mice	The ALF adjuvant conjugated with QS-21 induced the highest antibody titer with the highest IgG2c titers. Also, it augmented the number of activated B-cells compared to Montanide.	[138]
HBsAg	QS-21 liposome	Swine	The study demonstrated that the HBsAg formulated with QS-21 liposomes in dissolvable microneedle arraypatches induced similar immunization to the commercial HBsAg formulation.	[139]
Inactivated Bovine Viral Diarrhea Virus	QB-90 and IMXQB-90 (ISCOMs and *Q. brasiliensis* saponins	Mice	The use of *Q. brasiliensis* saponins in vaccine formulations either formulated in ISCOM structures or not promoted a satisfactory and promising immune response, similar to Quil-A®.	[140]

* FML: Fucose–Mannose-ligand antigen obtained from *Leishmania* (L.) *donovani* Sudan (LD 1S/MHOM/SD/00-strain 1S) antigen. ** LSP *Pb*CS 242–310: long synthetic polypeptide (LSP) PbCS 242–310, which represents C-terminus of circumsporozoite protein of *Plasmodium berghei*. *** HIV-1 DNA prime/protein–Env and Gag: polyvalent DNA prime/protein boost vaccine; **** LT(R192G): *Escherichia coli* heat-labile enterotoxin trypsin-cleavage site mutant; ***** ADAD System: includes *Q saponaria* saponins and/or the hydroalcoholic extract of *P. leucotomos* (PAL), emulsified with Montanide ISA763A as a water/oil (30/70) emulsion ⬪ LTB-ESAT-6: Plant-made antigen [transgenic *Arabidopsis thaliana* plants expressing the immuno-dominant tuberculosis antigen ESAT-6 fused to the B subunit of the *Escherichia*. ⬪⬪ GPI-0100: semi synthetic analogue of *Quillaja* Saponin; ⬪⬪⬪ sQS-21 (synthetic QS-21): mixture of QS-21-Api and QS-21-Xyl (65:35); ⬪⬪⬪⬪ AD-472 human herpes simplex virus (HSV) type 2 attenuated; ⬪⬪⬪⬪⬪ MF59: emulsion consisting of squalene–oil–water adjuvant; ^#^ MATRIX-M: mix of two mono saponin cages to which an antigen is added; ^##^ TA-CIN: recombinant *Human papillomavirus* type 16 (HPV16) L2, E6, and E7 in a unique protein.

**Table 3:** Clinical trials using *Quillaja* saponins as an adjuvant in vaccine formulations.

Antigen	Adjuvant	Objective	Findings	Ref.
CHO-derived gp120 protein from HIV-1	QS-21 or Al(OH)_3_	Evaluate safety and assess kinetics of immune response	Improved T cell response inducing CTL activation with T helper cells was observed in formulations with QS-21.	[149]
Globo H-KLH (carbohydrate antigen found in most breast cancer cells)	QS-21	Determine the formulation toxicity against cancerous cells, its immune response, and if the conjugation of Globo H with KLH would affect the immune response	Increase in specific antibody production and increased cytotoxicity either from complement system or antibody signaling.	[145]
PolySA and NP-polySA both conjugated with KLH (protein that in adults is associated with small cell lung cancer)	QS-21	Determine the immune response after vaccination and assess the impact of polySA chemical manipulation	NP-polySA vaccination resulted in higher antibody titers with IgM response. The IgM was reactive to small cell lung cancer.	[146]
MUC-2G/Globo H-KLH	GPI-0100⬪; GPI-0100-P ⬪⬪; QS-21	Present the use of GPI-0100 in humans and determine the safety and immunogenicity of a vaccine with different doses of GPI-0100.	GPI-0100 (5000 μg) and QS-21 (100 μg) produced comparable antibody titers. All adjuvanted vaccine doses were well tolerated and antigen-specific antibody titers matched increasing dose levels.	[83]
SL *	AS02A ⬪⬪⬪	Evaluate the potential of an AS02A adjuvanted formulation in healthy individuals and compare it to a non-adjuvanted vaccine.	The adjuvanted vaccine induced more significative humoral and Th1 immune responses compared with the non-adjuvanted formulation. Despite the AS02A recipients reporting local and general reactions more frequently than the non-adjuvanted group, the safety profile was acceptable.	[153]
FMP1 **	AS02A (oil-in-water formulation with mono- phosphoryl lipid A and QS-21	Evaluate the safety and immunogenicity of malaria vaccine FMP1/AS02A in adults	FMP1 formulated with AS02A was well tolerated and showed high immunogenicity with specific anti-MSP-142 antibody titers boosted and prolonged due to the vaccination.	[147]
Influenza	QS-21	Evaluate QS21 as an adjuvant and compare it with standard trivalent inactivated influenza	Local pain and post vaccination myalgias were greater in individuals that received QS-21. Despite increased serum antibodies, the mean titers for formulations (with or without QS-21) were not different.	[148]
RTS,S (recombinant proteins from *P. falciparum*)	AS02D	Evaluate the immunogenicity of RTS,S/AS02D formulation (liquid and lyophilized)	Lyophilized formulation is as efficient and safe as the liquid formulation, promoting satisfactory immunization.	[154]
P*f*AMA1 ***	Alhydrogel^TM^, Montanide ISA720 and AS02 Adjuvant System	Evaluate the immunogenicity and safety of P*f*AMA1 antigen (two different doses with three different adjuvants)	All formulations caused different reactogenicity with no serious reported adverse effects. All formulations tested induced antibody production, with AS02 being the most pronounced.	[155]
RTS,S ****	AS02A	Determine the safety of the RTS,S antigen formulated with AS02A adjuvant.	The formulation was well tolerated in children, with a good safety profile within the number of doses. The AS02A formulation caused fewer serious adverse events compared to the control group.	[156]
FMP2.1 *****	AS02A	Evaluate the reactogenicity, safety, and immunogenicity of the malaria vaccine FMP2.1/AS02A in adults.	The formulation was well tolerated and showed good safety. Also, it was highly immunogenic.	[157]
No antigen was used	*Quillaja* saponins	Determine if dietary QS can modify macrophage activity and investigate its effects on liver function and inflammatory response	An increase in chemotactic and phagocytosis activities were observed. Furthermore, no adverse effects were seen since no significant changes in immunoglobulin, transaminase, IL-1α, and TNF- α were observed.	[144]
NP-polySA-KLH (Polysialic acid conjugated to keyhole limpet hemocyanin (KLH))	QS-21	Confirm the safety profile and determine the optimal dose	The lowest optimal immunogenic dose was 10 µg, which resulted in consistent high-titer antibody responses.	[151]
MAGE-A3 (tumor-specific protein usually expressed in melanoma)	AS02B or AS15	Discover which adjuvant would cause a more robust and persistent immune response	AS15 provided a more robust immune response by activating more dendritic and B cells.	[152]
HBsAg (*Hepatitis B virus* surface antigen)	AS02B, AS02V, or AS01B	Evaluate the duration of humoral and cellular responses	All formulations induced persistent T CD4+ and CD8+ specific response, as well as B-cell response, indicating immunological memory.	[150]
Unimolecular conjugated Globo-H, GM2, sTn, TF, and Tf (markers usually expressed on ovarian cancer cell-surface)	QS-21	Evaluate safety and immunogenicity of the pentavalent synthetic vaccine	83% of individuals responded to at least three antigens with satisfactory immune response.	[158]
HBsAg (surface antigen of *Hepatitis B virus*), ovalbumin, CSP (*P. falciparum* circumsporozoite protein), and Varicella zoster glycoprotein E	AS01	Investigate how combining immune-stimulants results in innate immune response	AS01 triggers innate response, such as NK-cells, and activates CD8 T-cells in the lymph nodes, depending on macrophage, IL-12, and IL-18.	[159]

⬪ GPI-0100: Semi synthetic analogue of *Quillaja* saponin; ⬪⬪ GPI-0100P: GPI-0100 purified; ⬪⬪⬪ AS02A: oil-in-water formulation containing monophosphoryl lipid A and QS-21; * SL: recombinant hepatitis B protein containing the small protein (S) and the modified large (L) protein of the hepatitis B viral envelope, containing pre-Sl and pre-S2 sequences in addition to the entire S sequence. ** FMP1 (Falciparum malaria protein 1): recombinant protein based on the carboxy-terminal end of merozoite surface protein-1 (MSP-142) from the 3D7 clone of *P. falciparum*; *** P*f*AMA1: *Plasmodium falciparum* Apical Membrane Antigen; **** RTS,S: *Plasmodium falciparum* circumsporozoite surface antigen; ***** FMP2.1: recombinant protein (FMP2.1) based on Apical Membrane Antigen-1 (AMA-1) from the 3D7 clone of *P. falciparum.*
